# Supplementary material for: Multiple forest attributes underpin the supply of multiple ecosystem services
Source: Nat Commun. 2018 Nov 16;9:4839. doi: 10.1038/s41467-018-07082-4 (PMC6240034; doi:10.1038/s41467-018-07082-4)
Supplement: Supplementary file 1 — Supplementary Information [file 41467_2018_7082_MOESM1_ESM.pdf]

Multiple forest attributes underpin the provision of multiple ecosystem services

Felipe-Lucia et al.

Supplementary Information: list of material provided

Supplementary Figure 1. Relationship between forest attributes and forest management types: managed conifer forests (M_C), managed broadleaved forests (M_B), and unmanaged broadleaved forests (U_B).

Supplementary Figure 2. Relationship between ecosystem services and forest management types: managed conifer forests (M_C), managed broadleaved forests (M_B), and unmanaged broadleaved forests (U_B).

Supplementary Figure 3. Pairwise Spearman correlations among all potential drivers of ecosystem services considered for the analyses (i.e. the initial 21 forest attributes plus the four environmental factors).

Supplementary Figure 4. Change in Pearson correlations between ecosystem services due to a) the effect of environmental factors (E – All factors) and b) the effect of forest attributes (F – All factors).

Supplementary Figure 5. Significant effects found in simplified models (see Fig. 3 and Supplementary Table 6) plotted using unscaled data.

Supplementary Table 1. Description of the environmental factors and the forest attributes measured for 150 forest plots.

Supplementary Table 2. Description of the ecosystem service proxies assessed for 150 forest plots, including their category according to MEA (2005), units, year of collection, and sample size (N).

Supplementary Table 3. a) Variance partitioning of ecosystem services using multiple R^2. b) Variance partitioning of ecosystem services using adjusted R^2. c) AICc of the models used for the variance partitioning analyses.

Supplementary Table 4. a) Tests for Pearson correlation matrices comparison between ecosystem services including all factors and after subsequently removing the effects of environmental factors (E) and forest attributes (E+F). b) Tests to assess the effect of particular sets of ecosystem services in the matrices comparison.

Supplementary Table 5. Estimated overall effects of forest attributes on ecosystem services obtained from full linear models.

Supplementary Table 6. Estimated effects of 12 forest attributes on 14 ecosystem services obtained from simplified linear models.

Supplementary Table 7. a) Comparison of the effects of conifer species identity in full linear models. b) Comparison of the effects of broadleaved species identity in full linear models. c) Comparison of the effects removing overlapping species for wild edible plant species and shrubs richness and evenness in simplified models. d) Comparison of the effects removing overlapping species for wild edible plant species and shrubs richness and evenness in full models.

Supplementary Table 8. R^2, adjusted R^2, F-statistics and degrees of freedom (DF) for each of the 14 ecosystem service models.

Supplementary Table 9. List of edible fungi species identified in the 150 forest plots.

Supplementary Table 10. List of wild edible plants species for potential gathering found in the 150 forest plots and their average percentage of cover across plots.

Supplementary Table 11. List of plant species of special interest (i.e. of cultural value) found in the 150 forest plots and their average percentage of cover across plots.

Supplementary Methods.

Supplementary References.

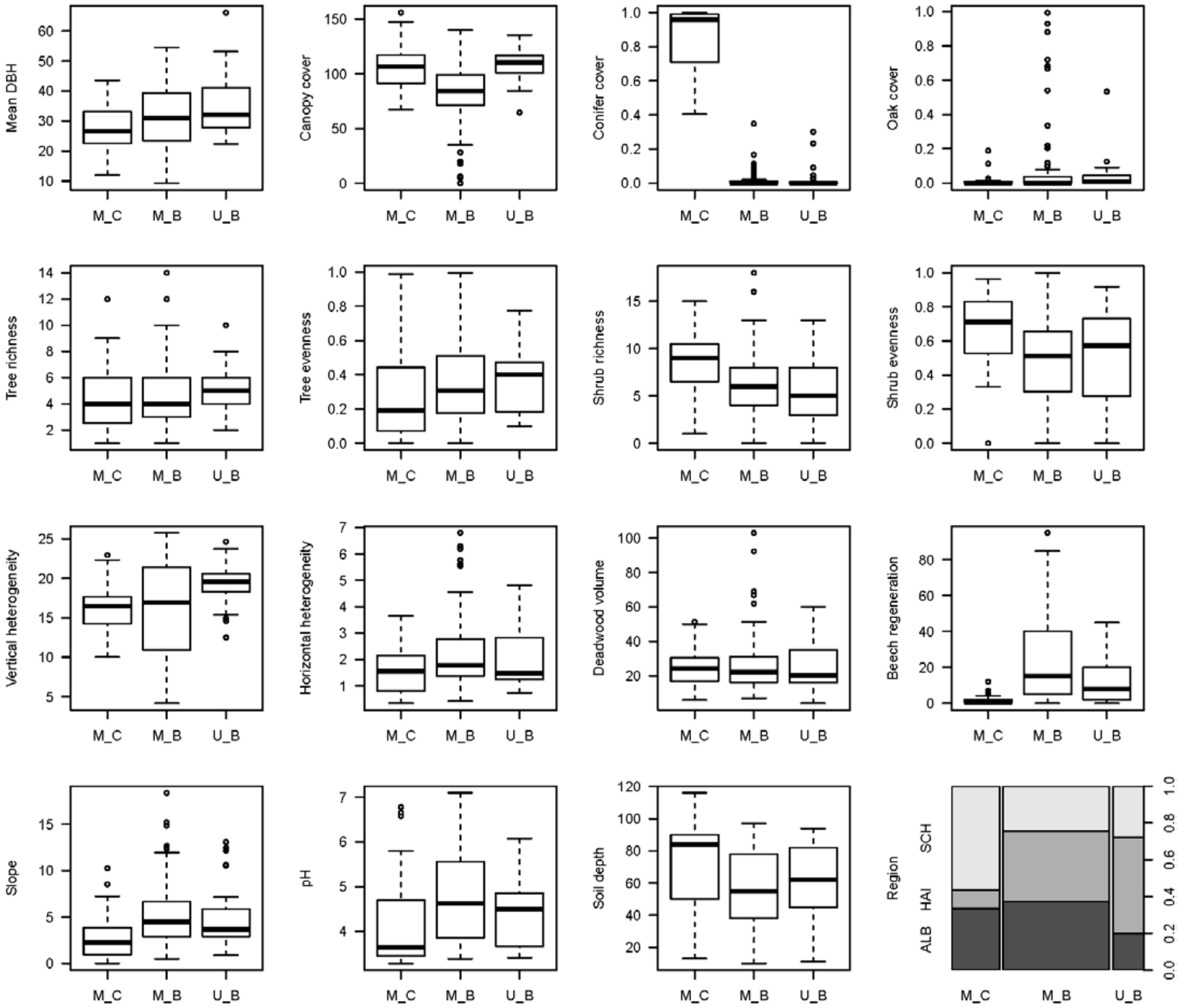

Supplementary Figure 1. Relationship between forest attributes and forest management types: managed conifer forests (M_C), managed broadleaved forests (M_B), and unmanaged broadleaved forests (U_B). Region: ALB (Schwäbische Alb), HAI (Hainich-Dün), SCH (Schorfheide-Chorin). Units as Supplementary Table 1 except for Region that shows proportions.

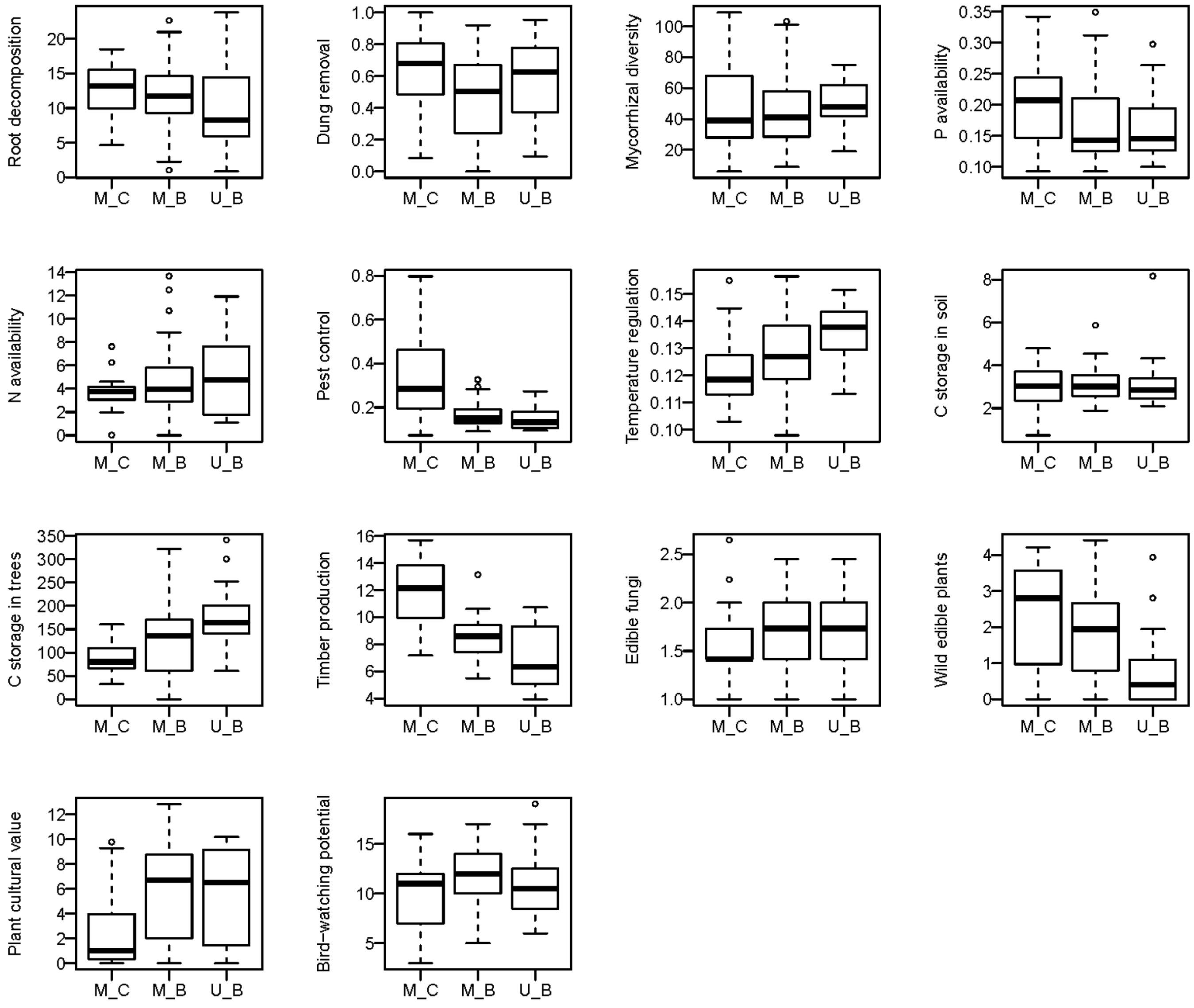

Supplementary Figure 2. Relationship between ecosystem services and forest management types: managed conifer forests (M_C), managed broadleaved forests (M_B), and unmanaged broadleaved forests (U_B). Units as Supplementary Table 2.

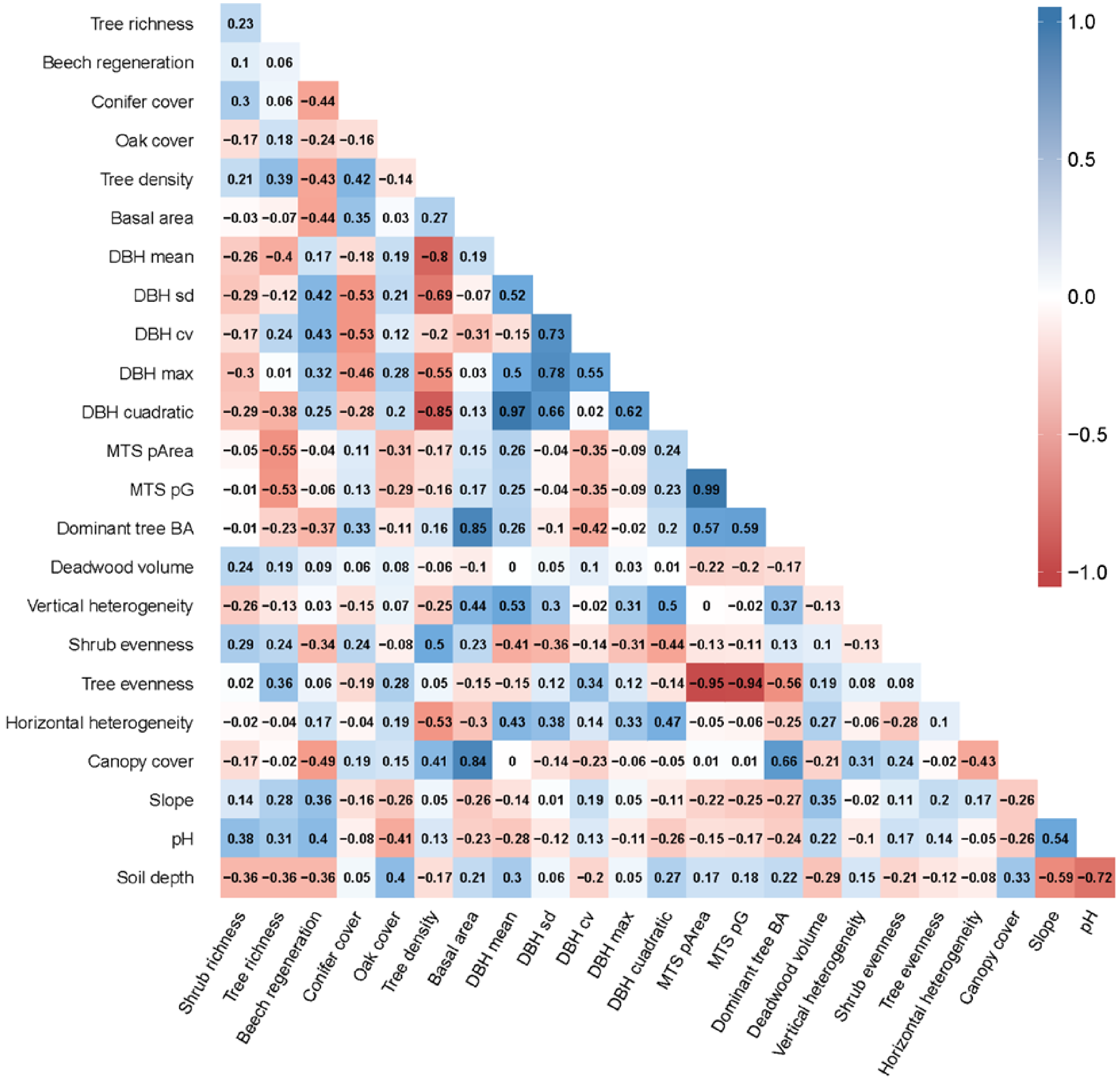

Supplementary Figure 3. Pairwise Spearman correlations among all potential drivers of ecosystem services considered for the analyses (i.e. the initial 21 forest attributes plus the four environmental factors). See Supplementary Table 1 for a description of the variables.

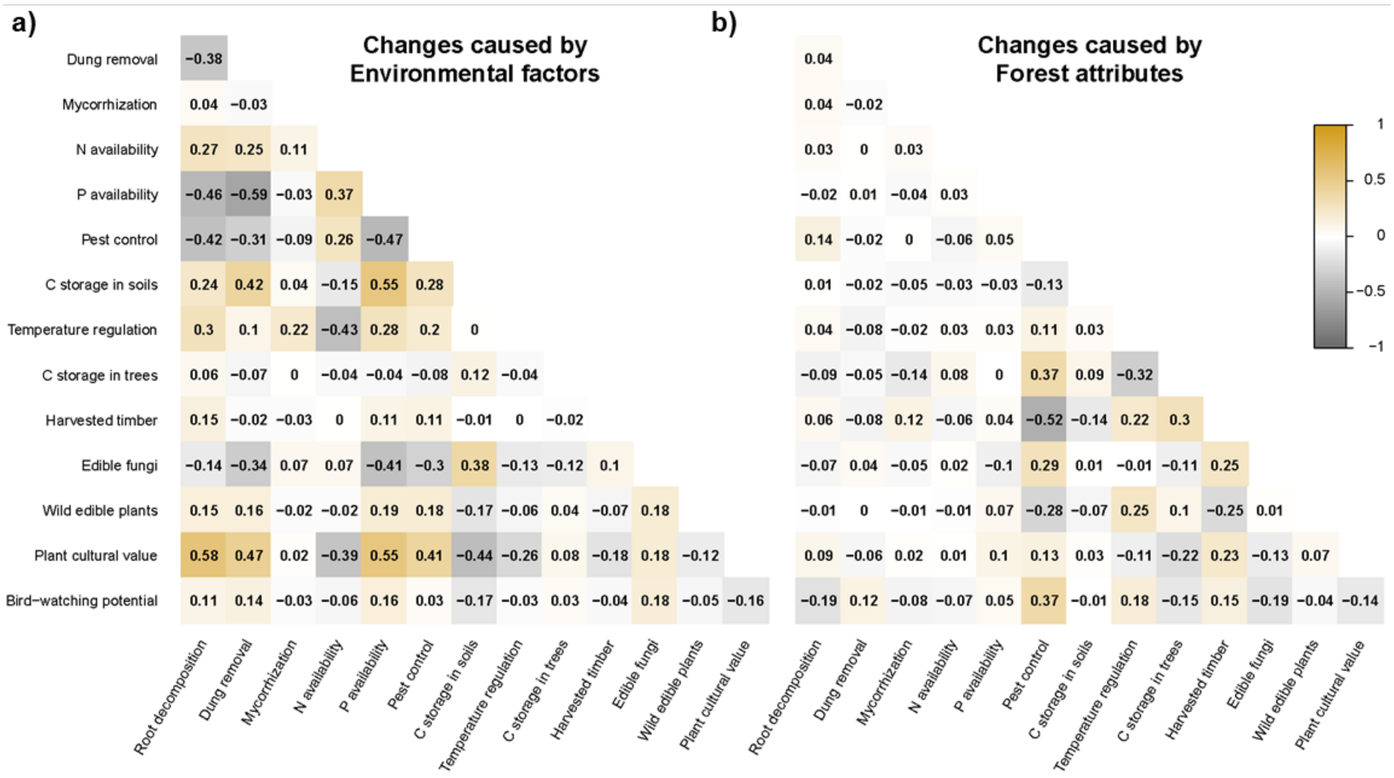

Supplementary Figure 4. Change in Pearson correlations between ecosystem services due to a) the effect of environmental factors (E – All factors) and b) the effect of forest attributes (F – All factors).

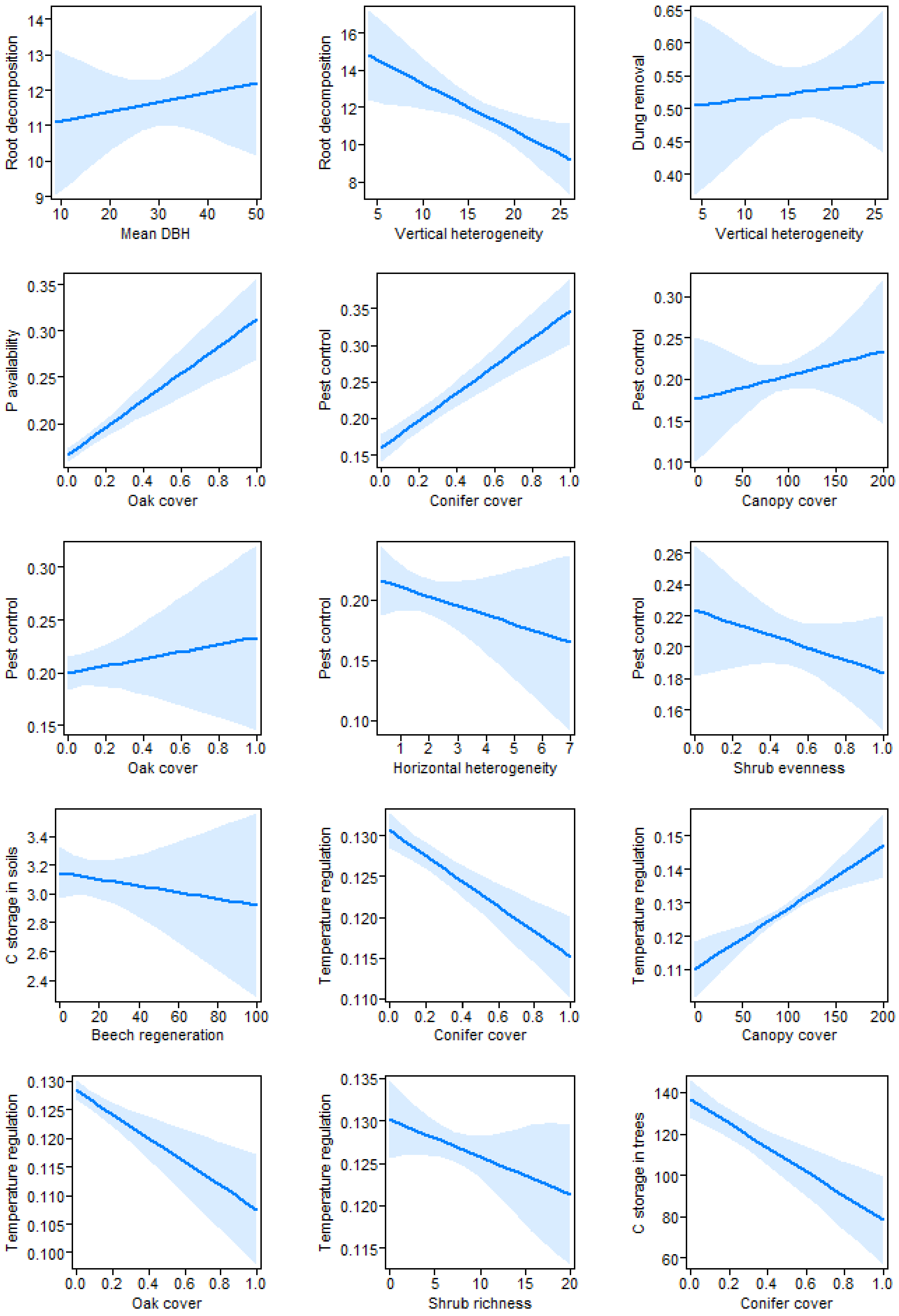
Root decomposition
9 10 11 12 13 14
10 20 30 40 50
Mean DBH
Root decomposition
8 10 12 14 16
5 10 15 20 25
Vertical heterogeneity
Dung removal
0.40 0.45 0.50 0.55 0.60 0.65
5 10 15 20 25
Vertical heterogeneity
P availability
0.20 0.25 0.30 0.35
0.0 0.2 0.4 0.6 0.8 1.0
Oak cover
Pest control
0.15 0.20 0.25 0.30 0.35
0.0 0.2 0.4 0.6 0.8 1.0
Conifer cover
Pest control
0.10 0.15 0.20 0.25 0.30
0 50 100 150 200
Canopy cover
Pest control
0.15 0.20 0.25 0.30
0.0 0.2 0.4 0.6 0.8 1.0
Oak cover
Pest control
0.10 0.15 0.20
1 2 3 4 5 6 7
Horizontal heterogeneity
Pest control
0.16 0.18 0.20 0.22 0.24 0.26
0.0 0.2 0.4 0.6 0.8 1.0
Shrub evenness
C storage in soils
2.4 2.6 2.8 3.0 3.2 3.4
0 20 40 60 80 100
Beech regeneration
Temperature regulation
0.110 0.115 0.120 0.125 0.130
0.0 0.2 0.4 0.6 0.8 1.0
Conifer cover
Temperature regulation
0.11 0.12 0.13 0.14 0.15
0 50 100 150 200
Canopy cover
Temperature regulation
0.100 0.105 0.110 0.115 0.120 0.125 0.130
0.0 0.2 0.4 0.6 0.8 1.0
Oak cover
Temperature regulation
0.115 0.120 0.125 0.130 0.135
0 5 10 15 20
Shrub richness
C storage in trees
60 80 100 120 140
0.0 0.2 0.4 0.6 0.8 1.0
Conifer cover

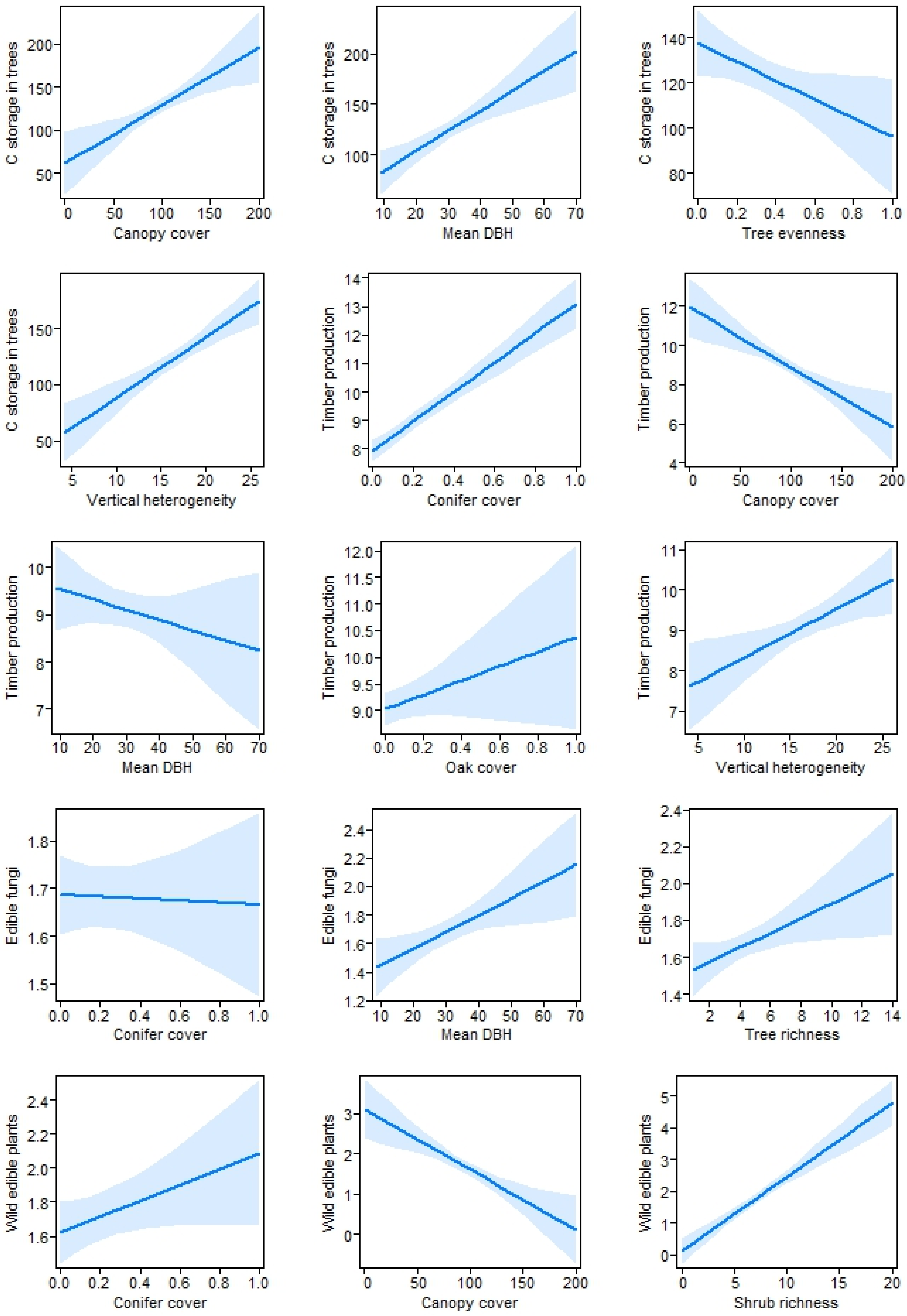

C storage in trees
200
150
100
50
0 50 100 150 200
Canopy cover
C storage in trees
200
150
100
10 20 30 40 50 60 70
Mean DBH
C storage in trees
140
120
100
80
0.0 0.2 0.4 0.6 0.8 1.0
Tree evenness
C storage in trees
150
100
50
5 10 15 20 25
Vertical heterogeneity
Timber production
14
13
12
11
10
9
8
0.0 0.2 0.4 0.6 0.8 1.0
Conifer cover
Timber production
12
10
8
6
4
0 50 100 150 200
Canopy cover
Timber production
10
9
8
7
10 20 30 40 50 60 70
Mean DBH
Timber production
12.0
11.5
11.0
10.5
10.0
9.5
9.0
0.0 0.2 0.4 0.6 0.8 1.0
Oak cover
Timber production
11
10
9
8
7
5 10 15 20 25
Vertical heterogeneity
Edible fungi
1.8
1.7
1.6
1.5
0.0 0.2 0.4 0.6 0.8 1.0
Conifer cover
Edible fungi
2.4
2.2
2.0
1.8
1.6
1.4
1.2
10 20 30 40 50 60 70
Mean DBH
Edible fungi
2.4
2.2
2.0
1.8
1.6
1.4
2 4 6 8 10 12 14
Tree richness
Wild edible plants
2.4
2.2
2.0
1.8
1.6
0.0 0.2 0.4 0.6 0.8 1.0
Conifer cover
Wild edible plants
3
2
1
0
0 50 100 150 200
Canopy cover
Wild edible plants
5
4
3
2
1
0
0 5 10 15 20
Shrub richness

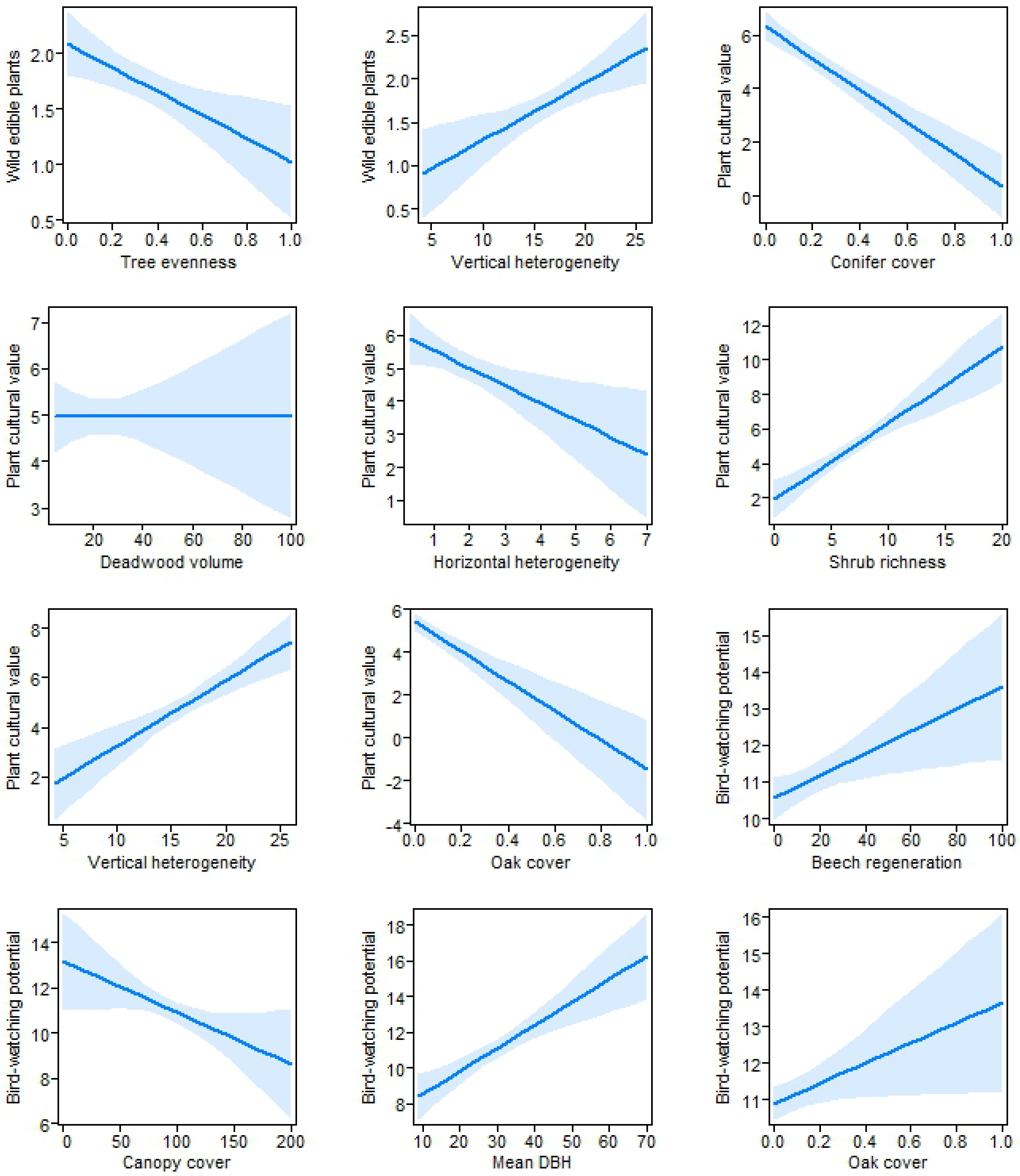

Supplementary Figure 5. Significant effects found in simplified models (see Fig. 3 and Supplementary Table 6) plotted using unscaled data. See Supplementary Table 1 for a description of the forest attributes and Supplementary Table 2 for a description of the ecosystem services.

Supplementary Table 1. Description of the environmental factors and the forest attributes measured for 150 forest plots. (DBH: diameter at breast height).

	**Variable**	**Description**	**Units**
Environment	Location	Biodiversity Exploratory site	Unitless
	Slope	Average slope of each plot, based on DTM.	Degrees
	Soil depth	Thickness of the mineral soil layer.	cm
	Soil pH	pH measurement of the mineral soil (0-10 cm).	Unitless
Structure	Canopy cover	Percentage of the plot covered by the projected tree (DBH >7 cm) crown.	%
	Mean DBH	Mean tree (DBH >7 cm) DBH.	cm
Composition	Conifer cover	Proportion of non-native or planted conifer species (*Abies alba, Larix sp., Picea abies, Pinus sylvestris, Pseudotsuga menziesii*) on total tree (DBH >7 cm) cover.	%
	Oak cover	Proportion of *Quercus* species on total tree (DBH >7 cm) cover.	%
Heterogeneity	Vertical heterogeneity	Number of layers in the vertical axis.	Number of layers
	Horizontal heterogeneity	Standard deviation of canopy height, related to the stand patchiness.	m^2 ha^{-1}
Diversity	Tree richness	Richness of tree species (DBH >7 cm).	Number of species
	Tree evenness	Evenness of tree species (DBH >7 cm).	Evenness of species
	Shrubs richness	Richness of the woody vegetation (<5 m).	Number of species
	Shrubs evenness	Evenness of the woody vegetation (<5 m).	Evenness of species
Regeneration	Beech regeneration	Cover of beech <5m.	%
Deadwood	Deadwood volume	Total deadwood volume, indicates habitat availability for wood decomposers.	m^3 ha^{-1}
Additional variables initially considered	*Tree density*	*Number of trees (DBH >7 cm).*	*trees* ha^{-1}
	Basal area	*Total area covered by tree (DBH >7 cm) stems.*	m^2 ha^{-1}
	DBH sd	*Standard deviation of tree (DBH >7 cm) DBH.*	*cm*
	DBH cv	*Coefficient of variation tree (DBH >7 cm) DBH.*	*%*
	DBH max	*Maximum tree (DBH >7 cm) DBH.*	*cm*
	DBH cuadratic	*Quadratic mean tree (DBH >7 cm) DBH.*	*cm*
	MTS pArea	*Proportion of the dominant tree species (DBH >7 cm) on covered ground area.*	*%*
	MTS pG	*Proportion of the dominant tree species (DBH >7 cm) on total basal area.*	*%*
	Dominant tree BA	*Total area covered by stems of the dominant tree species (DBH >7 cm).*	m^2 ha^{-1}

Supplementary Table 2. Description of the ecosystem service proxies assessed for 150 forest plots, including their category according to MEA (2005), units, year of collection, and sample size (N). (OTU: operational taxonomic unit).

**Category**	**Variable**	**Description**	**Units**	**Year**	**N**
Supporting	Root decomposition	Root litter mass loss rate	%	2012	135
	Dung removal	Dung removal rate	%	2014	150
	Mycorrhizal diversity	Richness of mycorrhizal OTU	Number of OTU	2011	111
	P availability	Content of extractable phosphorus in soil	g P kg^{-1} soil	2014	150
	N availability	Potential nitrification	ng NO_2 g^{-1} soil h^{-1}	2014	149
Regulating	Pest control	ratio (Predators + Parasitoids) / (Bark beetles)	Unitless	2010	149
	Temperature regulation	Temperature diurnal range	$°C^{-1}$	2011	150
	C storage in soil	Carbon stock in topsoil	kg C m^{-2} soil	2014	145
	C storage in trees	Carbon stored in trees	kg C m^{-2}	2012	150
Provisioning	Timber production	Mean annual increment in wood volume (Periodic annual increment for uneven-aged and managed forests)	m^3 ha^{-1} a^{-1}	2016	150
Cultural	Edible fungi	Richness of edible fungi	Number of species	2011	143
	Wild edible plants	Cover of species for plant gathering	%	2011	150
	Plant cultural value	Cover of plants of cultural interest	%	2011	150
	Bird-watching potential	Richness of birds	Number of species	2011	148

Supplementary Table 3. a) Variance partitioning of ecosystem services using multiple R^2. The three first columns are the R^2 of models including only environmental variables (E), those with only forest attributes (F), and the full models (E + F). The next three columns are the exclusive effects of the environmental variables, the forest attributes, and the shared variances between both. The last column is the unexplained variance for each ecosystem service modeled. The average variance explained across all ecosystem services (ES) is also given.

Ecosystem services	R^2 E	R^2 F	R^2 E + F	Exclusive E	Exclusive F	Shared E + F	Unexplained
Root decomposition	0.513	0.319	0.554	0.235	0.041	0.279	0.446
Dung removal	0.539	0.327	0.571	0.244	0.031	0.296	0.429
P availability	0.690	0.522	0.745	0.223	0.055	0.467	0.255
N availability	0.625	0.277	0.656	0.379	0.032	0.245	0.344
Mycorrhizal diversity	0.101	0.166	0.206	0.040	0.105	0.061	0.794
Pest control	0.357	0.488	0.701	0.214	0.344	0.143	0.299
Temperature regulation	0.382	0.496	0.673	0.177	0.291	0.205	0.327
C storage in soil	0.485	0.277	0.528	0.251	0.043	0.235	0.472
C storage in trees	0.098	0.678	0.691	0.013	0.593	0.085	0.309
Timber production	0.193	0.536	0.621	0.084	0.428	0.109	0.379
Edible fungi	0.363	0.295	0.554	0.259	0.191	0.103	0.446
Wild edible plants	0.143	0.595	0.600	0.005	0.457	0.138	0.400
Plant cultural value	0.646	0.620	0.774	0.153	0.128	0.492	0.226
Bird-watching potential	0.040	0.408	0.445	0.037	0.405	0.003	0.555
Average all ES	*0.370*	*0.429*	*0.594*	*0.165*	*0.225*	*0.204*	*0.406*

b) Variance partitioning of ecosystem services using adjusted R^2. The three first columns are the adjusted R^2 of models including only environmental variables (E), those with only forest attributes (F), and the full models (E + F). The next three columns are the exclusive effects of E, F, and the shared variances between both (E + F). The last column is the unexplained variance for each ecosystem service modeled. The average variance explained across all ecosystem services (ES) is also given.

Ecosystem services	Adjusted R^2 E	Adjusted R^2 F	Adjusted R^2 E + F	Exclusive E	Exclusive F	Shared E + F	Unexplained
Root decomposition	0.494	0.251	0.488	0.237	-0.006	0.258	0.512
Dung removal	0.523	0.267	0.514	0.248	-0.009	0.276	0.486
P availability	0.679	0.480	0.712	0.232	0.033	0.447	0.288
N availability	0.611	0.212	0.610	0.399	-0.001	0.212	0.390
Mycorrhizal diversity	0.058	0.062	0.058	-0.004	0.000	0.062	0.942
Pest control	0.334	0.442	0.662	0.220	0.328	0.114	0.338
Temperature regulation	0.360	0.451	0.630	0.179	0.270	0.181	0.370
C storage in soil	0.467	0.211	0.464	0.253	-0.003	0.214	0.536
C storage in trees	0.067	0.649	0.651	0.001	0.584	0.065	0.349
Harvested timber	0.165	0.495	0.571	0.076	0.406	0.089	0.429
Edible fungi	0.339	0.229	0.492	0.264	0.153	0.076	0.508
Wild edible plants	0.114	0.559	0.548	-0.011	0.434	0.125	0.452
Plant cultural value	0.633	0.587	0.744	0.158	0.111	0.476	0.256
Bird-watching potential	0.006	0.355	0.372	0.017	0.366	-0.011	0.628
Average all ES	*0.346*	*0.375*	*0.537*	*0.162*	*0.190*	*0.185*	*0.463*

c) AICc of the models used for the variance partitioning analyses. The three first columns are the AICc of the models including only environmental variables (E), those with only forest attributes (F), and the full models (E + F).The AICc of a null model is also given for comparison. The smallest AICc for each model is highlighted in bold.

	AICc E	AICc F	AICc E+F	AICc Null
Root decomposition	**299.844**	358.379	315.368	386.201
Dung removal	**323.159**	391.254	337.447	428.760
Mycorrhizal diversity	**317.271**	322.556	331.218	318.111
N availability	**286.959**	396.620	301.638	420.248
P availability	263.785	341.848	**261.447**	428.760
Pest control	370.892	323.253	**256.796**	425.923
C storage in soils	**328.970**	389.567	341.570	414.573
Temperature regulation	367.374	349.768	**298.632**	428.760
C storage in trees	423.983	**282.821**	289.399	428.760
Timber production	407.303	337.936	**320.950**	428.760
Edible fungi	355.241	380.733	**329.110**	408.899
Wild edible plants	416.256	**314.510**	325.412	428.760
Plant cultural value	283.883	306.326	**242.537**	428.760
Bird-watching potential	427.750	**362.568**	365.865	423.085

Supplementary Table 4. a) Tests for Pearson correlation matrices comparison between ecosystem services including all factors and after subsequently removing the effects of environmental factors (E) and forest attributes (E+F). The first column is the comparison test, the next three columns correspond to the unscaled Chi-square (χ^2) test statistic, and the next three give the Comparative Fit Index (CFI), Tucker-Lewis Index (TLI) and McDonald Measure of Centrality (Mc). Significant differences in χ^2, CFI and TLI values below 0.95, and Mc values below 0.90 indicate significant differences between matrices. See Methods for details.

Comparison	χ^2 diff	DF	Pr(>χ^2)	CFI	TLI	Mc
E *vs* All factors	138.411	91	0.001	0.942	0.884	0.924
E+F *vs* All factors	228.351	91	0.000	0.804	0.608	0.795
E+F *vs* E	98.013	91	0.289	0.975	0.949	0.988

b) Tests to assess the effect of particular sets of ecosystem services in the matrices comparison. Matrices are Pearson correlations between ecosystem services including all factors and after subsequently removing the effects of environmental factors (E) and forest attributes (E+F). The first column is the comparison test, the next three columns correspond to the unscaled Chi-square (χ^2), and the next three give the Comparative Fit Index (CFI), Tucker-Lewis Index (TLI) and McDonald Measure of Centrality (Mc) after dropping a set of ES (significant differences in χ^2, CFI and TLI values below 0.95, and Mc values below 0.90 indicate significant differences between matrices). The last column shows the number of times each comparison was significantly different for at least three of the indices after randomly dropping the same number of ecosystem services than the set assessed 1000 times.

	Comparison	χ^2 diff	DF	Pr(>χ^2)	CFI	TLI	MC	Significant random tests
Dropping supporting services (n=5)	E *vs* All factors	58.159	36	0.011	0.951	0.902	0.964	49
	E+F *vs* All factors	119.125	36	0.000	0.745	0.491	0.870	995
	E+F *vs* E	81.963	36	0.000	0.764	0.529	0.926	0
Dropping regulating services (n=4)	E *vs* All factors	89.249	45	0.000	0.872	0.744	0.929	37
	E+F *vs* All factors	139.360	45	0.000	0.725	0.450	0.854	959
	E+F *vs* E	35.440	45	0.846	1.000	1.210	1.000	0
Dropping provisioning services (n=1)	E *vs* All factors	130.959	78	0.000	0.929	0.858	0.915	0
	E+F *vs* All factors	189.836	78	0.000	0.826	0.652	0.829	1000
	E+F *vs* E	63.204	78	0.888	1.000	1.133	1.000	0
Dropping cultural services (n=4)	E *vs* All factors	97.662	45	0.000	0.887	0.774	0.916	37
	E+F *vs* All factors	138.626	45	0.000	0.776	0.553	0.855	959
	E+F *vs* E	57.769	45	0.096	0.914	0.828	0.979	0

Supplementary Table 5. Estimated overall effects of forest attributes on ecosystem services obtained from full linear models. DF = Degrees of freedom. DBH = Diameter at breast height. Significance levels: ***p ≤0.001; **p ≤0.01; *p ≤0.05.

	Estimate	Std. Error	DF	t value	Pr(>|t|)	Significance
Intercept	-0.001	0.017	1987	-0.036	0.971	
Shrub richness	0.047	0.026	1987	1.834	0.067	
Tree richness	0.030	0.024	1987	1.262	0.207	
Beech regeneration	0.018	0.023	1987	0.792	0.429	
Conifer cover	0.077	0.031	1987	2.456	0.014	*
Oak cover	0.021	0.022	1987	0.927	0.354	
Mean DBH	0.062	0.029	1987	2.157	0.031	*
Deadwood volume	-0.003	0.022	1987	-0.124	0.901	
Vertical heterogeneity	0.099	0.027	1987	3.733	0.000	***
Shrub evenness	0.006	0.024	1987	0.244	0.808	
Tree evenness	-0.005	0.021	1987	-0.231	0.817	
Horizontal heterogeneity	0.018	0.025	1987	0.701	0.483	
Canopy cover	-0.007	0.027	1987	-0.259	0.795	

Supplementary Table 6. Estimated effects of 12 forest attributes on 14 ecosystem services obtained from simplified linear models. Only those predictors retained after performing model simplification based on AIC are shown. Standard errors and *p*-values are also shown.

Ecosystem service	Predictor	Estimate	Std. Error	Pr(>|t|)
Root decomposition	Intercept	0.014	0.059	0.817
Root decomposition	Mean DBH	0.200	0.074	0.008
Root decomposition	Vertical heterogeneity	-0.154	0.071	0.032
Dung removal	Intercept	-0.002	0.055	0.968
Dung removal	Vertical heterogeneity	0.124	0.058	0.033
P availability	Intercept	0.003	0.044	0.944
P availability	Oak cover	0.168	0.049	0.001
Pest control	Intercept	-0.061	0.052	0.244
Pest control	Conifer cover	0.424	0.074	0.000
Pest control	Horizontal heterogeneity	-0.286	0.068	0.000
Pest control	Canopy cover	-0.158	0.068	0.023
Pest control	Shrub evenness	0.178	0.062	0.005
Pest control	Oak cover	-0.143	0.061	0.021
C storage in soils	Intercept	0.001	0.060	0.987
C storage in soils	Beech regeneration	-0.153	0.063	0.017
Temperature regulation	Intercept	-0.004	0.049	0.933
Temperature regulation	Shrub richness	-0.137	0.067	0.041
Temperature regulation	Oak cover	-0.180	0.061	0.004
Temperature regulation	Conifer cover	-0.250	0.076	0.001
Temperature regulation	Canopy cover	0.538	0.059	0.000
C storage in trees	Intercept	0.004	0.049	0.941
C storage in trees	Conifer cover	-0.292	0.064	0.000
C storage in trees	Mean DBH	0.358	0.066	0.000
C storage in trees	Canopy cover	0.225	0.065	0.001
C storage in trees	Vertical heterogeneity	0.374	0.072	0.000
C storage in trees	Tree evenness	-0.150	0.054	0.006
Timber production	Intercept	0.020	0.054	0.713
Timber production	Canopy cover	-0.219	0.073	0.003
Timber production	Conifer cover	0.829	0.074	0.000
Timber production	Mean DBH	-0.163	0.073	0.027
Timber production	Vertical heterogeneity	0.199	0.080	0.014
Timber production	Oak cover	0.172	0.065	0.009
Edible fungi	Intercept	0.011	0.059	0.851
Edible fungi	Tree richness	0.260	0.072	0.000
Edible fungi	Conifer cover	-0.249	0.073	0.001
Edible fungi	Mean DBH	0.246	0.072	0.001
Wild edible plants	Intercept	0.002	0.053	0.965
Wild edible plants	Shrub richness	0.621	0.072	0.000
Wild edible plants	Conifer cover	0.193	0.079	0.015
Wild edible plants	Vertical heterogeneity	0.247	0.065	0.000
Wild edible plants	Tree evenness	-0.189	0.057	0.001
Wild edible plants	Canopy cover	-0.298	0.069	0.000
Plant cultural value	Intercept	-0.003	0.041	0.944
Plant cultural value	Shrub richness	0.296	0.055	0.000
Plant cultural value	Conifer cover	-0.404	0.061	0.000
Plant cultural value	Oak cover	-0.123	0.049	0.013

Plant cultural value	Deadwood volume	-0.096	0.047	0.044
Plant cultural value	Vertical heterogeneity	0.200	0.045	0.000
Plant cultural value	Horizontal heterogeneity	-0.109	0.049	0.028
Bird-watching potential	Intercept	0.004	0.065	0.952
Bird-watching potential	Beech regeneration	0.179	0.075	0.018
Bird-watching potential	Oak cover	0.290	0.070	0.000
Bird-watching potential	Mean DBH	0.439	0.072	0.000
Bird-watching potential	Canopy cover	-0.202	0.074	0.007

Supplementary Table 7. a) Comparison of the effects of conifer species identity in full linear models. Standard errors, *p*-values and adjusted *p*-values are also shown.

Ecosystem service	Predictor	Estimate	Std. Error	Pr(>|t|)	Adj. Pr(>|t|)
Root decomposition	Conifer cover	0.016	0.109	0.886	0.988
Root decomposition	Spruce cover	0.188	0.174	0.287	0.842
Root decomposition	Pine cover	-0.008	0.172	0.962	0.989
Dung removal	Conifer cover	0.089	0.102	0.385	0.792
Dung removal	Spruce cover	0.483	0.208	0.025	0.414
Dung removal	Pine cover	0.022	0.257	0.933	0.981
Mycorrhizal diversity	Conifer cover	0.100	0.168	0.553	0.918
Mycorrhizal diversity	Spruce cover	0.442	0.376	0.251	0.842
Mycorrhizal diversity	Pine cover	-0.563	0.400	0.170	0.552
N availability	Conifer cover	0.161	0.094	0.089	0.356
N availability	Spruce cover	0.043	0.151	0.776	0.935
N availability	Pine cover	-0.145	0.220	0.515	0.853
P availability	Conifer cover	0.001	0.079	0.986	0.988
P availability	Spruce cover	-0.055	0.154	0.722	0.935
P availability	Pine cover	-0.290	0.243	0.242	0.629
Pest control	Conifer cover	0.318	0.079	0.000	0.000
Pest control	Spruce cover	-0.147	0.159	0.362	0.891
Pest control	Pine cover	0.729	0.298	0.020	0.250
C storage in soils	Conifer cover	0.155	0.109	0.159	0.482
C storage in soils	Spruce cover	0.242	0.278	0.389	0.908
C storage in soils	Pine cover	0.206	0.412	0.621	0.853
C storage in trees	Conifer cover	-0.241	0.087	0.006	0.061
C storage in trees	Spruce cover	-0.165	0.144	0.257	0.842
C storage in trees	Pine cover	-0.361	0.113	0.003	0.091
Temperature regulation	Conifer cover	-0.175	0.090	0.054	0.266
Temperature regulation	Spruce cover	0.092	0.202	0.650	0.935
Temperature regulation	Pine cover	-0.496	0.253	0.059	0.383
Timber production	Conifer cover	0.921	0.097	0.000	0.000
Timber production	Spruce cover	0.927	0.219	0.000	0.000
Timber production	Pine cover	-0.312	0.323	0.342	0.751
Edible fungi	Conifer cover	-0.158	0.108	0.145	0.481
Edible fungi	Spruce cover	-0.072	0.245	0.772	0.935
Edible fungi	Pine cover	-0.981	0.337	0.007	0.182
Wild edible plants	Conifer cover	0.191	0.098	0.054	0.266
Wild edible plants	Spruce cover	0.063	0.170	0.714	0.918
Wild edible plants	Pine cover	0.506	0.254	0.056	0.364
Plant cultural value	Conifer cover	-0.404	0.074	0.000	0.000
Plant cultural value	Spruce cover	-0.139	0.160	0.388	0.908
Plant cultural value	Pine cover	-0.042	0.180	0.817	0.928
Bird-watching potential	Conifer cover	0.074	0.115	0.521	0.878
Bird-watching potential	Spruce cover	-0.156	0.209	0.460	0.910
Bird-watching potential	Pine cover	-0.289	0.356	0.423	0.801

b) Comparison of the effects of broadleaved species identity in full linear models. Standard errors, *p*-values and adjusted *p*-values are also shown.

Ecosystem service	Predictor	Estimate	Std. Error	Pr(>|t|)	Adj. Pr(>|t|)
Root decomposition	Oak cover	0.114	0.146	0.437	0.810
Root decomposition	Beech cover	0.093	0.244	0.704	0.908
Dung removal	Oak cover	-0.191	0.117	0.104	0.474
Dung removal	Beech cover	-0.294	0.199	0.142	0.535
Mycorrhizal diversity	Oak cover	-0.003	0.194	0.987	1.000
Mycorrhizal diversity	Beech cover	0.553	0.338	0.107	0.477
N availability	Oak cover	0.198	0.112	0.081	0.397
N availability	Beech cover	0.438	0.193	0.026	0.170
P availability	Oak cover	0.212	0.077	0.007	0.084
P availability	Beech cover	0.115	0.131	0.381	0.754
Pest control	Oak cover	-0.150	0.056	0.009	0.084
Pest control	Beech cover	-0.248	0.096	0.012	0.098
C storage in soils	Oak cover	-0.184	0.118	0.122	0.498
C storage in soils	Beech cover	-0.313	0.199	0.119	0.498
Temperature regulation	Oak cover	-0.107	0.103	0.299	0.689
Temperature regulation	Beech cover	0.273	0.175	0.122	0.498
C storage in trees	Oak cover	-0.064	0.116	0.579	0.866
C storage in trees	Beech cover	-0.024	0.197	0.902	1.000
Timber production	Oak cover	0.104	0.094	0.274	0.647
Timber production	Beech cover	0.092	0.161	0.566	0.866
Edible fungi	Oak cover	0.213	0.106	0.048	0.277
Edible fungi	Beech cover	0.251	0.182	0.171	0.568
Wild edible plants	Oak cover	-0.031	0.121	0.800	0.996
Wild edible plants	Beech cover	-0.085	0.206	0.681	0.892
Plant cultural value	Oak cover	-0.196	0.086	0.024	0.162
Plant cultural value	Beech cover	-0.143	0.146	0.329	0.711
Bird-watching potential	Oak cover	0.271	0.138	0.052	0.289
Bird-watching potential	Beech cover	0.092	0.233	0.693	0.900

c) Comparison of the effects removing overlapping species for wild edible plant species and shrubs richness and evenness in simplified models.

Dataset	Ecosystem service	Predictor	Estimate	Std. Error	Pr(>|t|)
**Original**	Wild edible plants	Intercept	0.002	0.053	0.965
	Wild edible plants	Shrub richness	0.621	0.072	0.000
	Wild edible plants	Conifer cover	0.193	0.079	0.015
	Wild edible plants	Vertical heterogeneity	0.247	0.065	0.000
	Wild edible plants	Tree evenness	-0.189	0.057	0.001
	Wild edible plants	Canopy cover	-0.298	0.069	0.000
	Wild edible plants	Intercept	0.001	0.059	0.989
**Removing overlapping species**	Wild edible plants	Shrub richness	0.439	0.077	0.000
	Wild edible plants	Conifer cover	0.346	0.083	0.000
	Wild edible plants	Vertical heterogeneity	0.208	0.072	0.005
	Wild edible plants	Tree evenness	-0.180	0.064	0.005
	Wild edible plants	Canopy cover	-0.350	0.076	0.000

d) Comparison of the effects removing overlapping species for wild edible plant species and shrubs richness and evenness in full models.

Dataset	Ecosystem service	Predictor	Estimate	Std. Error	Pr(>|t|)	Adj. Pr(>|t|)
**Original**	Wild edible plants	Intercept	0.002	0.054	0.971	0.988
	Wild edible plants	Shrub richness	0.641	0.079	0.000	0.000
	Wild edible plants	Tree richness	-0.011	0.073	0.880	0.988
	Wild edible plants	Beech regeneration	-0.084	0.071	0.243	0.614
	Wild edible plants	Conifer cover	0.191	0.098	0.054	0.266
	Wild edible plants	Oak cover	0.033	0.070	0.644	0.948
	Wild edible plants	Mean DBH	0.062	0.089	0.486	0.842
	Wild edible plants	Deadwood volume	0.008	0.067	0.905	0.988
	Wild edible plants	Vertical heterogeneity	0.210	0.082	0.012	0.091
	Wild edible plants	Shrub evenness	-0.064	0.074	0.389	0.800
	Wild edible plants	Tree evenness	-0.186	0.064	0.005	0.051
	Wild edible plants	Horizontal heterogeneity	-0.033	0.079	0.677	0.974
	Wild edible plants	Canopy cover	-0.306	0.083	0.000	0.000
**Removing overlapping species**	Wild edible plants	Intercept	0.000	0.060	0.995	0.999
	Wild edible plants	Shrub richness	0.411	0.083	0.000	0.000
	Wild edible plants	Tree richness	0.069	0.080	0.392	0.779
	Wild edible plants	Beech regeneration	0.007	0.078	0.930	0.999
	Wild edible plants	Conifer cover	0.331	0.108	0.003	0.030
	Wild edible plants	Oak cover	0.045	0.079	0.575	0.908
	Wild edible plants	Mean DBH	0.146	0.098	0.137	0.475
	Wild edible plants	Deadwood volume	0.053	0.074	0.471	0.824
	Wild edible plants	Vertical heterogeneity	0.169	0.091	0.066	0.286
	Wild edible plants	Shrub evenness	0.086	0.091	0.342	0.732
	Wild edible plants	Tree evenness	-0.191	0.071	0.008	0.066
	Wild edible plants	Horizontal heterogeneity	-0.055	0.088	0.529	0.867
	Wild edible plants	Canopy cover	-0.368	0.091	0.000	0.000

Table 8. R^2, adjusted R^2, F-statistics and degrees of freedom (DF) for each of the 14 ecosystem service models. Metrics for both full and simplified models are shown.

	Full models				Simplified models			
**Ecosystem services**	**R^2**	**Adj. R^2**	**F-statistic**	**DF**	**R^2**	**Adj. R^2**	**F-statistic**	**DF**
Root decomposition	0.074	-0.019	0.799	120	0.060	0.046	4.178	130
Dung removal	0.079	-0.003	0.968	135	0.031	0.024	4.638	146
P availability	0.174	0.101	2.372	135	0.075	0.069	11.908	146
N availability	0.080	-0.003	0.959	132	0.000	0.000	-	-
Mycorrhizal diversity	0.117	0.006	1.056	96	0.000	0.000	-	-
Pest control	0.540	0.498	13.084	134	0.600	0.581	30.659	102
Temperature regulation	0.472	0.425	10.054	135	0.437	0.421	27.767	143
C storage in soil	0.092	0.009	1.104	130	0.041	0.034	5.840	138
C storage in trees	0.658	0.627	21.606	135	0.633	0.620	48.552	141
Timber production	0.530	0.488	12.687	135	0.514	0.496	28.998	137
Edible fungi	0.281	0.213	4.161	128	0.217	0.200	12.671	137
Wild edible plants	0.529	0.487	12.645	135	0.520	0.503	30.710	142
Plant cultural value	0.312	0.251	5.107	135	0.343	0.315	12.266	141
Bird-watching potential	0.424	0.372	8.165	133	0.397	0.379	22.071	134

Supplementary Table 9. List of edible fungi species identified in the 150 forest plots.

**Edible fungi species**
Amanita rubescens
Boletus edulis
Chlorophyllum olivieri
Cortinarius stillatitius
Hydnum rufescens
Hygrophorus discoideus
Hygrophorus poetarum
Laccaria amethystina
Laccaria laccata
Lycoperdon perlatum
Macrolepiota procera
Pluteus cervinus
Psathyrella piluliformis
Russula cyanoxantha
Russula decolorans
Russula heterophylla
Russula integra
Russula paludosa
Russula vesca
Russula virescens
Tricholoma columbetta
Tricholoma orirubens
Xerocomus pruinatus

Supplementary Table 10. List of wild edible plants species for potential gathering found in the 150 forest plots and their average percentage of cover across plots.

Wild edible plant species	Cover (%)
Aegopodium podagraria	0.353
Allium ursinum	0.597
Corylus avellana	0.177
Fragaria vesca	0.727
Galium odoratum	3.321
Hypericum hirsutum	0.057
Hypericum maculatum	0.023
Hypericum montanum	0.023
Hypericum perforatum	0.052
Juniperus communis	0.007
Malus sp.	0.009
Prunus avium	0.044
Prunus padus	0.020
Prunus spinosa	0.047
Pyrus communis	0.003
Ribes alpinum	0.003
Ribes uva-crispa	0.020
Rubus fruticosus-corylifolius agg.	1.341
Rubus idaeus	4.200
Sambucus nigra	0.561
Sorbus aria	0.037
Sorbus aucuparia	0.267
Sorbus torminalis	0.003
Vaccinium myrtillus	0.331

Supplementary Table 11. List of plant species of special interest (i.e. of cultural value) found in the 150 forest plots and their average percentage of cover across plots.

Plant species of cultural value	Cover (%)
Alliaria petiolata	0.094
Allium ursinum	0.597
Anemone nemorosa	20.549
Anemone ranunculoides	0.780
Arum maculatum	0.121
Asarum europaeum	1.120
Campanula patula	0.003
Campanula rotundifolia	0.003
Campanula trachelium	0.043
Cardamine bulbifera	0.980
Cephalanthera damasonium	0.025
Cephalanthera rubra	0.007
Chrysosplenium alternifolium	0.007
Colchicum autumnale	0.003
Convallaria majalis	0.044
Daphne mezereum	0.062
Digitalis purpurea	0.001
Epilobium angustifolium	0.075
Euphorbia amygdaloides	0.040
Euphorbia cyparissias	0.054
Galium odoratum	3.321
Geum urbanum	0.270
Helleborus foetidus	0.011
Hepatica nobilis	0.017
Lamium galeobdolon agg.	2.177
Lathraea squamaria	0.007
Lathyrus vernus	0.231
Lilium martagon	0.028
Listera ovata	0.007
Maianthemum bifolium	0.014
Malus sp.	0.009
Mercurialis perennis	4.443
Paris quadrifolia	0.171
Polygonatum multiflorum	0.051
Polygonatum odoratum	0.001
Polygonatum verticillatum	0.144
Primula elatior veris agg.	0.132
Prunus avium	0.044
Pulmonaria obscura	0.093
Ranunculus ficaria	1.747
Ranunculus lanuginosus	0.040
Sorbus aria	0.037
Sorbus aucuparia	0.267
Sorbus torminalis	0.003
Viburnum lantana	0.003
Viburnum opulus	0.007
Vincetoxicum hirundinaria	0.003

Supplementary Methods.

Ecosystem service measurement

In each of the 150 forest plots we assessed proxies for 14 ecosystem services. Most soil ecosystem services (i.e., P availability, N availability, C storage in soils, mycorrhizal diversity and edible fungi) were sampled in a joint soil sampling campaign that collected 14 samples of the upper 10 cm of the mineral soil in each of 150 plots along two 40 m long transects using cores with a diameter of 5 cm. The joint soil sampling campaign took place in May 2011 and May 2014. In each campaign, the 14 soil samples were mixed into a composite sample before further analysis. Roots were used for the determination of the associated fungal species richness by pyrosequencing and identification of mycorrhizal fungi by a standard procedure (ITS2 sequencing and accepting species names at the 97 per cent level of identity with species in UNITE or NCBI databases)[1]. Cultural services based on plant records (i.e., wild edible plants and plant species of special cultural value) were sampled during a vegetation survey of a 400 m² subplot in each of the 150 plots in 2011. Cover of each plant species was estimated across four different vegetation layers (herbs, shrubs <5m, trees 5 – 10 m, trees > 10 m), and the sum of the cover per plant species across vegetation layers was used to estimate proxies of the ecosystem services described below.

Because the proxies for each of the different services represent different time frames, we have included data collected in the closest available dates (e.g. most soil, plants and temperature data were collected in 2011). In other cases, values were available to represent a rotation period (e.g. timber production). In any case, we consider these proxies as snap-shot measures of a particular time-period and acknowledge that values could change over time.

Description of the methods used to measure each ecosystem service proxy:

Root decomposition. Fine root decomposition plays an important role in element cycling in forest ecosystems[2]. We measured decomposition of fine roots (<2 mm) within the upper 10 cm of the mineral soil. Three polyester litterbags per plot, with a mesh size of 100 micrometers, were filled with fine roots collected from 2 year old European beech (*Fagus sylvatica* L.). These were buried in each of the 150 plots in October 2011 and were then harvested after 12 months in October 2012. We used the percentage of root litter mass loss as a proxy of decomposition[3].
Dung removal. Dung beetle communities contribute to the rapid decomposition of fecal deposits from both wild mammals and domestic livestock, representing a key ecosystem service[4]. We installed five dung piles (cow, sheep, horse, wild boar, red deer) on each 150 plots and collected the remaining dung after 48 hours. The average percentage of dung dry mass removed (mostly by tunneling dung beetles) was used as indicator of dung removal rates[4].
P availability. Phosphorus (P) can be a limiting element for plant growth and therefore can limit the functioning of the ecosystem[5,6]. We investigated the availability of P in forest soils by collecting soil samples as described above. Available P was extracted with 0.5M $NaHCO3$ (pH = 8.5) following the Olsen methodology[7] and measured with Inductively Coupled Plasma/Optical Emission and Spectrometry (ICP-OES, PerkinElmer Optima 5300 DV, S10 auto sampler).
N availability. Nitrogen (N) can be a limiting element for plant growth and therefore can limit the functioning of the ecosystem[8,9]. We investigated the nitrification process in forest soils in terms of potential nitrification activity associated with N availability. Soil samples were collected during the joint campaign as described above. Potential nitrification measures were derived from the abundance of nitrifying bacteria following ref[10] and were used as a proxy of soil N availability[11,12].
Mycorrhizal diversity. Higher species richness of mycorrhizal communities has been associated with higher functional diversity[13] and therefore higher rates of water and nutrient uptake[13–15], pathogen protection[16], and enzyme production[17]. We sampled the fungal community associated with roots following the abovementioned description of the joint soil campaign. The number of mycorrhizal operational taxonomic units (OTU) was used as a proxy of mycorrhizal diversity.
Pest control. Pest control is an important forest ecosystem service that can have an effect on other services like production of quality timber and aesthetic value[18,19]. We assessed the abundance of potential pest species

on ambrosia beetles (i.e. bark beetles, an important pest in forests[20]), together with their antagonists. We selected ambrosia beetles within the tribe Xyleborini because they comprise generalist species that are all found in conifer as well as broadleaved forests with some showing preference either for broadleaved (e.g. *Trypodendron domesticum* (L. 1758)) or conifer (e.g. *Trypodendron lineatum* (Oliv. 1795)) trees. All species share antagonists among predators and parasitoids. We assessed bark beetle and antagonists abundance by pheromone traps using a bottle trap. Lineatin lures and ethanol were used as attractants for bark beetles and their antagonists. Traps were emptied every second day during the main flight activity of the beetles and in weekly to monthly intervals afterwards. We standardized the data based on the method proposed by[21]. We only considered bark beetles that are attracted by lineatin or ethanol (i.e. species within the tribe Xyleborini) and predators and parasitoids that are mentioned as antagonists of Xyleborini in the literature[22]. Predator-prey ratios have been frequently used as measure of pest control potential in different systems[23–25]. The ratio between the sum of predators and parasitoids *vs*. bark beetles was used as a proxy of pest control (i.e. (Predators + Parasitoids) / (Bark beetles)).

Temperature regulation. Forests buffer extreme temperatures due to canopy cover[26]. We collected data on air temperature 2 m above ground from climatic stations installed in each of the 150 plots. Diurnal temperature ranges (DTR) were calculated as differences between daily maximum and minimum temperature values[27]. Missing data was interpolated from surrounding stations. The inverse value of the average DTR per plot (DTR^{-1}) was used as indicator to facilitate the interpretation of the results, so higher values of the indicator mean higher temperature regulation of the forest plot[28].

C storage in soils. Forest soils contain important carbon pools. To estimate the amounts of carbon stored in forest topsoils, we followed the abovementioned description of the joint soil sampling campaign. An aliquot of <2 mm sieved soil was homogenized with a ball mill (RETSCH MM200, Retsch, Haan, Germany) and used to determine total C concentrations by dry combustion in an elemental analyser (VarioMax, Hanau, Germany). Inorganic carbon was determined after combustion of organic carbon at 450 °C for 16 h using the same elemental analyser and total organic carbon was afterwards determined from the difference between total and inorganic carbon. Organic carbon stocks were determined by multiplying organic carbon concentrations with the total soil mass (0-10 cm) per unit area (<2 mm) per m^2 at each site.

C storage in trees. Trees are also important carbon sinks, as they store carbon in their tissues via photosynthesis. To assess the amount of carbon stored in trees in each of the 150 plots, we estimated the living tree volume of each plot[29]. Dry biomass was estimated using the conversion factor of 0.43 for plots dominated by spruce, 0.49 for plots dominated by pine, 0.66 for plots dominated by oak and 0.68 for plots dominated by beech, according to ref[30]. The carbon stored in trees is approximately 50% of its dry biomass[31].

Timber production. Timber is one of the main products extracted from forests.

We quantified timber production of each 150 plots as mean annual increment (MAI) across rotation (i.e. culmination of MAI) for even-aged forests and as periodic annual increment (PAI) between two forest inventories for uneven-aged and unmanaged forests. MAI was estimated based on site class or site maps of forest administrations. Culmination of MAI is estimated on 70 years to 100 years for Picea abies, 70 years to 90 years for *Pinus sylvestris*, 110 years to 130 for *Quercus* sp., and 160 years for *Fagus sylvatica*. PAI was estimated as the difference between the increment measured during the first forest inventory (2008 - 2011) and the second forest inventory (2015 - 2016) of our plots divided by the time span in years. All values are given as volume above bark (> 7 cm in diameter) in $m^3\ ha^{-1}\ a^{-1}$.

Edible fungi. Mushroom collection or observation is common in forests, and is an important recreational activity considered as a cultural service[32,33]. We estimated the potential of our forests to harbor edible fungi by analyzing fungal species pools in forest soils following the abovementioned description of the joint soil campaign. Edible fungi were identified following the criteria of the German Mycological Society, excluding those species with inconsistent edible value[34]. We used species richness of the edible fungi as a proxy of potential observation of edible fungi (See complete list in Supplementary Table 9).

Wild edible plants. A recreational use of the forests is the collection of fruits, nuts, berries and medicinal plants for non-commercial use[35]. We estimated the potential recreational interest of forest plots following the methods for plant-based cultural services described above. Based on expert knowledge, we identified wild edible plant species known to be collected in the forest plots. Total cover of these species was used as a proxy of potential gathering of wild edible plants (See complete list in Supplementary Table 10).

Plants of cultural value. Forests harbor a great variety of plants, many of which are of special interest for botanists and education, such as the forest specialists *Helleborus spp., Asarum europaeum,* or *Galium odoratum*[36], or are species blooming in early spring appreciated for their aesthetic value[37], such as *Anemone nemorosa* and *Allium ursinum*. We recorded all plant species following the abovementioned method

description for plant-based cultural services. Plant species of special interest for the general public or for botanists were identified by botanists from the Botanical Society of Bern (Bernische Botanische Gesellschaft) with knowledge on people preferences. Total cover of these species was used as a proxy of potential plant cultural value (See complete list in Supplementary Table 11).
Bird-watching potential. Forests provide recreational opportunities for those interested in bird-watching. To estimate the bird-watching potential of forests plots, we performed five bird surveys during breeding times (March to June 2011) counting the number of individuals seen or heard during 5 minutes at the center of each plot[38]. Bird species richness was used as a proxy for bird watching potential.

Forest attributes
We performed forest inventories from 2008 to 2011 to measure forest attributes in each of the 150 plots. We first selected 21 forest attributes, which represent the variation in forest management between stands and which we can expect to be linked to our ecosystem service measures. These included several measures of structure, composition, heterogeneity, diversity, regeneration and deadwood content of the forests (Supplementary Fig. 3). To avoid fitting correlated explanatory variables we tested for multi-collinearity based on variance inflation factors (VIF) using the corvif function in R[39], with a threshold of 3[40]. After this procedure, 12 forest attributes were selected, which represent unique stand characteristics related to structure (mean DBH, canopy cover), composition (conifer and oak cover), heterogeneity (vertical and horizontal), diversity (tree richness and evenness, shrub richness and evenness), regeneration of native species (beech regeneration) and deadwood volume.

Description of the methods used to measure each of the 12 forest attributes selected:

Canopy cover. The percentage of each plot covered by tree crowns was estimated from the total crown projection area of all trees (DBH >7 cm) in a plot.
Mean diameter at breast height (DBH). Average DBH of the plot (only trees with DBH >7 cm were measured), which is closely related to the stand age.
Conifer cover. Percentage of total DBH comprised of non-native or planted conifer species (i.e. *Pinus sylvestris, Picea abies, Larix sp., Abies alba, Pseudotsuga menziesii,* as sorted by cover).
Oak cover. Percentage of total DBH comprised by all *Quercus* species. Including the cover of both oak and conifers in the analysis represents the variation in tree species composition between the stands. The effect of conifer cover represents the difference between broadleaved and conifer dominated stands and the effect of oak cover additionally represents the difference between beech and oak dominated stands (note that we could not additionally include beech cover directly in the models as it would be highly negatively correlated with oak and conifer cover).
Vertical heterogeneity. The number of layers in the vertical axis of each forest plot was measured using terrestrial LiDAR laser scans. We used a laser-beam density of 44.4 Million beams per scan to obtain 3D point clouds using Faro Focus 3D (Faro Technologies Inc., Lake Marry, USA). We performed nine scans per plot, systematically distributed in the centre, east, north-east, north, north-west, west, south-west, south and south-east, respectively. The resulting three-dimensional point clouds were stratified into layers of one meter thickness. We calculated the inverse of the Simpson index as an indicator of forest vertical heterogeneity using the number of canopy layers that are effectively occupied by foliage and woody component from the total number of layers[41]. Larger index values reflect a more even occupation of layers by foliage along the vertical axis as well as greater stand height.
Horizontal heterogeneity. Standard deviation of canopy height using 25 cells, each of 400 m^2 per hectare (i.e., per plot). We used an airborne full-wave Riegl LMS-Q 560 scanner (Riegl Laser Measurement Systems GmbH, Horb, Austria), with a sampling accuracy of 50 cm for the LiDAR data[42]. This indicator characterises horizontal heterogeneity in terms of patchiness without being sensitive to fine-grained heterogeneity of the canopy surface due to cell size.
Tree richness. We estimated the number of tree species, from individuals above 5 m high, based on vegetation surveys performed in 400 m^2 of each of the 150 plots in 2011.
Tree evenness. We estimated the evenness of tree species, above 5 m height, based on vegetation surveys performed in 400 m^2 plots in 2011. Pielou's index (*J'*) was used to calculate a measure of evenness uncorrelated with species richness, which ranges between 0 and 1.
Shrub richness. We estimated the species richness of the woody vegetation below 5 m in height, based on

vegetation surveys performed in 400 m² plots in 2011.
Shrub evenness. We estimated the Pielou evenness (*J'*) of the woody vegetation below 5 m in height, based on vegetation surveys performed in 400 m² plots in 2011.
Beech regeneration. We estimated the cover of beech (*Fagus sylvatica*) below 5 m in height based on vegetation surveys performed in 400 m² plots in 2011.
Deadwood volume. We measured total deadwood volume, including downed and standing items larger than 25 cm and stumps larger than 7cm[29].

Environmental factors
Location. Each of the three regions covered by the Biodiversity Exploratories. This factor includes regional effects of climate, land use history, soil texture and elevation, amongst others.
Slope. We estimated the slope of each of the 150 plots using second-order finite differences[43,44] based on digital terrain models (DTM) and averaging values for each plot[42].
Soil depth. We determined soil depth by sampling a soil core in the centre of all 150 plots in 2009. We used a motor-driven soil-column cylinder with a diameter of 8.3 cm for the soil sampling (Eijkelkamp, Giesbeek, The Netherlands). The combined thickness of all topsoil and subsoil horizons was used as a proxy of soil depth.
Soil pH. We collected soil samples following the abovementioned description of the 2011 joint soil-sampling campaign. pH was determined as the average value of two measurements based on 0.01M $CaCl_2$ with a soil solution ratio of 1:2.5.

Supplementary References

1. Goldmann, K. *et al.* Divergent habitat filtering of root and soil fungal communities in temperate beech forests. *Sci. Rep.* **6,** (2016).
2. Hobbie, S. E. Effects of plant species on nutrient cycling. *Trends Ecol. Evol.* **7,** 336–339 (1992).
3. Solly, E. F. *et al.* Factors controlling decomposition rates of fine root litter in temperate forests and grasslands. *Plant Soil* **382,** 203–218 (2014).
4. Frank, K., Hülsmann, M., Assmann, T., Schmitt, T. & Blüthgen, N. Land use affects dung beetle communities and their ecosystem service in forests and grasslands. *Agric. Ecosyst. Environ.* **243,** 114–122 (2017).
5. Vitousek, P. M., Porder, S., Houlton, B. Z. & Chadwick, O. A. Terrestrial phosphorus limitation: mechanisms, implications, and nitrogen–phosphorus interactions. *Ecol. Appl.* **20,** 5–15 (2010).
6. Lang, F. *et al.* Soil phosphorus supply controls P nutrition strategies of beech forest ecosystems in Central Europe. *Biogeochemistry* **136,** 5–29 (2017).
7. Olsen, S. R., Cole, C. V., Watanabe, F. S. & Dean, L. A. *Estimation of available phosphorus in soils by extraction with sodium bicarbonate.* **939:1–19.,** (Gov. Printing Office, 1954).
8. Vitousek, P. M. & Howarth, R. Nitrogen limitation on land and in the sea: how can it occur? Biogeochemistry 13:87-115. *Biogeochemistry* **13,** 87–115 (1991).
9. LeBauer, D. S. & Treseder, K. K. Nitrogen Limitation of Net Primary Productivity in Terrestrial Ecosystems Is Globally Distributed. *Ecology* **89,** 371–379 (2008).
10. Hoffmann, H., Schloter, M. & Wilke, B.-M. Microscale-scale measurement of potential nitrification rates of soil aggregates. *Biol. Fertil. Soils* **44,** 411–413 (2007).
11. Allan, E. *et al.* Land use intensification alters ecosystem multifunctionality via loss of biodiversity and changes to functional composition. *Ecol. Lett.* **18,** 834–843 (2015).
12. Soliveres, S. *et al.* Biodiversity at multiple trophic levels is needed for ecosystem multifunctionality. *Nature* **536,** 456–459 (2016).
13. Courty, P.-E. *et al.* The role of ectomycorrhizal communities in forest ecosystem processes: New perspectives and emerging concepts. *Soil Biol. Biochem.* **42,** 679–698 (2010).

14. Pena, R. & Polle, A. Attributing functions to ectomycorrhizal fungal identities in assemblages for nitrogen acquisition under stress. *ISME J.* **8,** 321–330 (2014).

15. Pena, R., Tejedor, J., Zeller, B., Dannenmann, M. & Polle, A. Interspecific temporal and spatial differences in the acquisition of litter-derived nitrogen by ectomycorrhizal fungal assemblages. *New Phytol.* **199,** 520–528 (2013).

16. Graham, J. H. What do root pathogens see in mycorrhizas? *New Phytol.* **149,** 357–359 (2001).

17. Buée, M., Courty, P.-E., Mignot, D. & Garbaye, J. Soil niche effect on species diversity and catabolic activities in an ectomycorrhizal fungal community. *Soil Biol. Biochem.* **39, 39,** (2007).

18. Jactel, H. *et al.* The influences of forest stand management on biotic and abiotic risks of damage. *Ann. For. Sci.* **66,** 701–701 (2009).

19. Bengtsson, J. Biological control as an ecosystem service: partitioning contributions of nature and human inputs to yield: Ecosystem services and human inputs. *Ecol. Entomol.* **40,** 45–55 (2015).

20. Raffa, K. F. *et al.* Cross-scale Drivers of Natural Disturbances Prone to Anthropogenic Amplification: The Dynamics of Bark Beetle Eruptions. *BioScience* **58,** 501–517 (2008).

21. Grégoire, J.-C., Piel, F., Proft, M. D. & Gilbert, M. Spatial Distribution of Ambrosia-Beetle Catches: A Possibly Useful Knowledge to Improve Mass-Trapping. *Integr. Pest Manag. Rev.* **6,** 237–242 (2001).

22. Kenis, M., Wermelinger, B. & Grégoire, J.-C. Research on Parasitoids and Predators of Scolytidae – A Review. in *Bark and Wood Boring Insects in Living Trees in Europe, a Synthesis* 237–290 (Springer, Dordrecht, 2007). doi:10.1007/978-1-4020-2241-8_11

23. Klein, A.-M., Steffan-Dewenter, I. & Tscharntke, T. Predator–prey ratios on cocoa along a land-use gradient in Indonesia. *Biodivers. Conserv.* **11,** 683–693 (2002).

24. Bianchi, F. J. J. A., Schellhorn, N. A. & Cunningham, S. A. Habitat functionality for the ecosystem service of pest control: reproduction and feeding sites of pests and natural enemies. *Agric. For. Entomol.* **15,** 12–23 (2013).

25. Werf, W. van der, Nyrop, J. P. & Hardman, J. M. Sampling predator/prey ratios to predict cumulative pest density in the mite - predatory mite system Panonychus ulmi - Typhlodromus pyri in apples. *Asp. Appl. Biol.* **37,** 41–51 (1994).

26. Frey, S. J. K. *et al.* Spatial models reveal the microclimatic buffering capacity of old-growth forests. *Sci. Adv.* **2,** e1501392–e1501392 (2016).

27. Scheitlin, K. N. & Dixon, P. G. Diurnal Temperature Range Variability due to Land Cover and Airmass Types in the Southeast. *J. Appl. Meteorol. Climatol.* **49,** 879–888 (2009).

28. Felipe-Lucia, M. R., Comín, F. A. & Bennett, E. M. Interactions Among Ecosystem Services Across Land Uses in a Floodplain Agroecosystem. *Ecol. Soc.* **19,** (2014).

29. Kahl, T. & Bauhus, J. An index of forest management intensity based on assessment of harvested tree volume, tree species composition and dead wood origin. *Nat. Conserv.* **7,** 15–27 (2014).

30. Lohmann, U. *Holz-Handbuch*. (DRW-Verlag, 2012).

31. Intergovernmental Panel on Climate Change (IPCC). Good Practice Guidance for Land Use, Land-Use Change and Forestry. (2003).

32. CICES. Towards a common classification of ecosystem services. CICES V4.3. (2016).

33. Millennium Ecosystem Assessment. *Ecosystems and Human Well-Being: Our Human Planet: Summary for Decision Makers*. (Island Press, 2005).

34. Deutsche Gesellschaft für Mykologie e.V. Speisepilze. (2015). Available at: https://www.dgfm-ev.de/speise-und-giftpilze/Speisepilze. (Accessed: 9th April 2018)

35. Gamfeldt, L. *et al.* Higher levels of multiple ecosystem services are found in forests with more tree species. *Nat. Commun.* **4,** 1340 (2013).

36. Schmidt, M., Kriebitzsch, W.-U. & Ewald, J. *Waldartenlisten der Farn- und Blütenpflanzen, Moose und Flechten Deutschlands*. (Bundesamt für Naturschutz (BfN), 2011).

37. Bhattacharya, D. K., Ghosh, A. & de Castro, F. Cultural Services. *Ecosyst. Hum. Well- Policy Responses Find. Responses Work. Group* **3,** 401 (2005).

38. Renner, S. C. *et al.* Temporal Changes in Randomness of Bird Communities across Central Europe. *PLOS ONE* **9,** e112347 (2014).

39. R Development Core Team. *R: A language and environment for statistical computing.* (R Foundation for Statistical Computing, 2016).

40. Zuur, A. F. *Mixed effects models and extensions in ecology with R*. (Springer, 2009).

41. Ehbrecht, M., Schall, P., Juchheim, J., Ammer, C. & Seidel, D. Effective number of layers: A new measure for quantifying three-dimensional stand structure based on sampling with terrestrial LiDAR. *For. Ecol. Manag.* **Complete,** 212–223 (2016).

42. Nieschulze, J., Zimmermann, R., Börner, A. & Schulze, E. D. An assessment of forest canopy structure by LiDAR: Derivation and stability of canopy structure parameters across forest management types. *Forstarchiv* **83,** 195–209 (2012).

43. Ritter, P. A vector-based slope and aspect generation algorithm. *Photogramm. Eng. Remote Sens.* **53,** 1109–1111 (1987).

44. Zhou, Q. & Liu, X. Analysis of errors of derived slope and aspect related to DEM data properties. *Comput. Geosci.* **30,** 369–378 (2004).
