# Supplementary material for: Multiple forest attributes underpin the supply of multiple ecosystem services
Source: Nat Commun. 2018 Nov 16;9:4839. doi: 10.1038/s41467-018-07082-4 (PMC6240034; doi:10.1038/s41467-018-07082-4)
Supplement: Supplementary file 8 — Description of Additional Supplementary Files [file 41467_2018_7082_MOESM8_ESM.pdf]

Description of Additional Supplementary Files

Supplementary Data 1a. Estimated effects of the four environmental factors (slope, pH, soil depth and location) on ecosystem services used for the variance partitioning analysis.
Supplementary Data 1b. Estimated effects of the 12 forest attributes on ecosystem services used for the variance partitioning analysis.
Supplementary Data 1c. Estimated effects of the four environmental factors (slope, pH, soil depth and location) and the 12 forest attributes on ecosystem services used for the variance partitioning analysis.
Supplementary Data 2. Test of significant differences between correlation matrices for each pair of ecosystem services.
Supplementary Data 3. Estimated effects of 12 forest attributes on 14 ecosystem services obtained from full linear models.

Supplementary Information: contains supplementary figures, tables, and methods (with references).

List of material provided

Supplementary Figure 1. Relationship between forest attributes and forest management types: managed conifer forests (M_C), managed broadleaved forests (M_B), and unmanaged broadleaved forests (U_B).

Supplementary Figure 2. Relationship between ecosystem services and forest management types: managed conifer forests (M_C), managed broadleaved forests (M_B), and unmanaged broadleaved forests (U_B).

Supplementary Figure 3. Pairwise Spearman correlations among all potential drivers of ecosystem services considered for the analyses (i.e. the initial 21 forest attributes plus the four environmental factors).

Supplementary Figure 4. Change in Pearson correlations between ecosystem services due to a) the effect of environmental factors (E – All factors) and b) the effect of forest attributes (F – All factors).

Supplementary Figure 5. Significant effects found in simplified models (see Fig. 3 and Supplementary Table 6) plotted using unscaled data.

Supplementary Table 1. Description of the environmental factors and the forest attributes measured for 150 forest plots.

Supplementary Table 2. Description of the ecosystem service proxies assessed for 150 forest plots, including their category according to MEA (2005), units, year of collection, and sample size (N).

Supplementary Table 3. a) Variance partitioning of ecosystem services using multiple R^2. b) Variance partitioning of ecosystem services using adjusted R^2. c) AICc of the models used for the variance partitioning analyses.

Supplementary Table 4. a) Tests for Pearson correlation matrices comparison between ecosystem services including all factors and after subsequently removing the effects of environmental factors (E) and forest attributes (E+F). b) Tests to assess the effect of particular sets of ecosystem services in the matrices comparison.

Supplementary Table 5. Estimated overall effects of forest attributes on ecosystem services obtained from full linear models.

Supplementary Table 6. Estimated effects of 12 forest attributes on 14 ecosystem services obtained from simplified linear models.

Supplementary Table 7. a) Comparison of the effects of conifer species identity in full linear models. b) Comparison of the effects of broadleaved species identity in full linear models. c) Comparison of the effects removing overlapping species for wild edible plant species and shrubs richness and evenness in simplified models. d) Comparison of the effects removing overlapping species for wild edible plant species and shrubs richness and evenness in full models.

Supplementary Table 8. R^2, adjusted R^2, F-statistics and degrees of freedom (DF) for each of the 14 ecosystem service models.

Supplementary Table 9. List of edible fungi species identified in the 150 forest plots.

Supplementary Table 10. List of wild edible plants species for potential gathering found in the 150 forest plots and their average percentage of cover across plots.

Supplementary Table 11. List of plant species of special interest (i.e. of cultural value) found in the 150 forest plots and their average percentage of cover across plots.

Supplementary Methods.

Supplementary References.
